# Implementation of a Statewide Fentanyl Possession Law and Opioid-Related Overdose Deaths

**DOI:** 10.1001/jamahealthforum.2025.2654

**Published:** 2025-08-01

**Authors:** Cole Jurecka, Joella Adams, Pranav Padmanabhan, Jason Glanz, Paul Christine, Xiaoyu Guan, Danielle Kline, Ingrid Binswanger, Joshua Barocas

**Affiliations:** 1 Division of General Internal Medicine, University of Colorado School of Medicine, Aurora; 2 RTI International, Durham, North Carolina; 3 Department of Epidemiology, Colorado School of Public Health, Aurora; 4 Institute for Health Research, Kaiser Permanente Colorado, Aurora; 5 Colorado Permanente Medical Group, Denver; 6 Department of Health Systems Science, Bernard J. Tyson Kaiser Permanente School of Medicine, Pasadena, California

## Abstract

**Importance:**

In 2022, Colorado passed legislation making possession of small amounts of fentanyl, a high-potency synthetic opioid, a felony. Whether the Colorado law affected opioid overdose fatalities and whether those effects differed by racial and ethnic subgroups is unknown.

**Objective:**

To estimate the association between the change in criminal penalties for fentanyl possession with opioid-related overdose deaths (OODs) in Colorado.

**Design, Setting, and Population:**

Serial cross-sectional study comparing OODs among adults (≥18 years) who died of an overdose and population estimates before and after Colorado House Bill (HB) 22-1326 was enacted in July 2022 (January 2018-November 2023) using autoregressive integrated moving averages (ARIMA) model time series forecasting. Monthly OOD rates per 100 000 residents were calculated using state population estimates from the American Community Survey 5-Year Data and the Colorado Department of Local Affairs State Demography Office. Overdose death rates were calculated separately by racial and ethnic group (Hispanic, non-Hispanic Black, and non-Hispanic White). Data were analyzed from January 2018 to 2023.

**Exposure:**

Enactment of HB 22-1326 changed the legal penalty for possession of any drug weighing 1 g to 4 g that contained any amount of fentanyl from a misdemeanor to a level-4 drug felony punishable by up to 180 days in jail and up to 2 years of probation.

**Main Outcome:**

The difference between expected and observed OOD rates following the enactment of increased criminal penalties.

**Results:**

A total of 7099 OODs were analyzed (1798 Hispanic [25.3%], 451 Non-Hispanic Black [6.4%], and 4170 Non-Hispanic White [58.7%], 680 other [9.5%] and not included in the race and ethnicity categories). OODs increased across the study period in Colorado from 20.46 per 100 000 adults in January 2018 to 37.78 per 100 000 adults in November 2023. Among different racial and ethnic groups, the non-Hispanic Black population had the highest increase in OODs (9.3 per 100 000 in 2018 to 56.9 per 100 000 in 2023) followed by the Hispanic population. There was no difference between the observed and expect overdose deaths for the overall population following the enactment of HB 22-1326. However, there were significant increases in 4 of 13 months after policy implementation among the non-Hispanic Black population.

**Conclusions and Relevance:**

The results of this serial cross-sectional study suggest that increased criminal penalties for fentanyl possession did not change preexisting trends of OODs in Colorado and may have been associated with an increase in opioid overdoses in the Black population. These results should be interpreted in light of increasing opioid overdose rates in Black populations nationally during the study period.

## Key Points

**Question:**

Did legislation making possession of small quantities of fentanyl a felony in Colorado reduce overdose fatalities, and did mortality differ by racial and ethnic groups?

**Findings:**

In this serial cross-sectional study, opioid-related overdose deaths (OODs) increased from 20.46 per 100 000 in January 2018 to 37.78 per 100 000 adults in November 2023. Among racial and ethnic groups, the non-Hispanic Black population had the highest increase in OODs (9.3 to 56.9 per 100 000) followed by the Hispanic population.

**Meaning:**

Increasing criminal penalties for fentanyl possession did not lower preexisting trends of OODs in Colorado and may be associated with an increase among the Black population.

## Teaser

This cross-sectional analysis compares trends in opioid overdose mortality in Colorado before and after July 2022 legislation increased fentanyl possession penalties and examines its effect on racial and ethnic subgroups.

## Introduction

High-potency synthetic opioids such as illicitly manufactured fentanyl and fentanyl analogues have been responsible for a growing proportion of opioid-related overdose deaths (OODs) from 2016 through 2023.^1,2^ State responses to this crisis have largely been aimed at fentanyl distribution and/or possession. In 2022, Colorado adopted House Bill (HB) 22-1326,^3^ which made possession of any drug weighing 1 g to 4 g that contained any amount of fentanyl a level-4 drug felony punishable by up to 180 days in jail and up to 2 years of probation. Prior to this law, possession of less than 4 g of fentanyl was considered a misdemeanor offense.

As more states consider increased criminalization, it is important to understand whether Colorado’s law resulted in a change in overdose deaths and the extent to which different subgroups were impacted. The objective of this analysis is to compare trends in opioid overdose mortality in Colorado before and after the legislation making possession of fentanyl a felony became effective in July 2022 and to examine any differences by racial and ethnic subgroups.

## Methods

This was a serial cross-sectional study of the adult (≥18 years) Colorado population that experienced an OOD from January 2018 to November 2023. This study was reported in concordance with the Strengthening the Reporting of Observational Studies in Epidemiology (STROBE) reporting guidelines for cohort studies. This study was approved by the Colorado Multiple Institutional Review Board.

An overdose is considered opioid-related if the individual’s death record included any of the multiple cause-of-death *International Statistical Classification of Diseases and Related Health Problems, Tenth Revision (ICD-10)* codes used by the Centers for Disease Control and Prevention (CDC) for opioid overdose classification. Specifically, these codes are T40.0 (opium), T40.1 (heroin), T40.2 (natural opioid analgesics), T40.3 (methadone), T40.4 (synthetic opioid analgesics other than methadone), and T40.6 (other and unspecified narcotics). We obtained monthly overdose data from the Colorado Department of Public Health and Environment. We excluded those who were younger than 18 years at the time of death, were residents of Colorado who experienced an overdose death outside of the state, and were nonresidents who experienced an overdose death in Colorado. We calculated monthly OOD rates per 100 000 residents, using state population estimates from the American Community Survey 5-Year Data and the Colorado Department of Local Affairs State Demography Office. We also calculated OOD rates separately by racial and ethnic group (Hispanic, non-Hispanic Black, and non-Hispanic White). Other racial and ethnic groups were excluded due to months with no recorded OODs.

We used state-level data on monthly OOD rates to fit autoregressive integrated moving average (ARIMA) models to predict overdose deaths from August 2022 through November 2023. We used predictions (based on trends before HB 22-1326 became law) to calculate the difference between predicted and observed OOD. ARIMA models are a type of time series analysis that can account for autocorrelation and seasonality.^4^ Generating forecasted values based on prior trends was guided by the Box-Jenkins method.^5^ We used trends in OOD rates from January 2018 onward to coincide with increases in synthetic OOD.^6^ We ran ARIMA models for the overall state population and performed additional analyses stratifying by race and ethnicity. Using an automated algorithm [*auto.arima(),* R] and model selection approaches in SAS, we selected final models with the lowest information criteria—ARIMA (0, 1, 1) for the overall adult population, ARIMA (3, 1, 2) for the Hispanic population, ARIMA(0, 1, 1) for the non-Hispanic Black population, and ARIMA (0, 1, 1) for the non-Hispanic White population. To assess the sensitivity of our results to the definition of OODs, we performed sensitivity analyses that included overdose deaths caused by all drug types.

Analyses were conducted in SAS version 9.4 (SAS Institute Inc) and R version 4.3.3.

## Results

From January 1, 2018, through November 30, 2023, a total of 7099 OODs in Colorado were analyzed (1798 Hispanic [25.3%], 451 Non-Hispanic Black [6.4%], and 4170 Non-Hispanic White [58.7%], 680 other [9.5%] and not included in the race and ethnicity categories). The overall yearly overdose rate in Colorado increased from 11.99 per 100 000 people in 2018 to 25.0 per 100 000 people in 2023, with a peak of 26.65 in 2021. Overdose trends by race and ethnicity were similar to overall trends except among the non-Hispanic Black and Hispanic populations, which were between 1.5 and 2 times higher than the non-Hispanic White population (Table).

**Table.** Yearly Overdose Counts and Rates by Race and Ethnicity in Colorado (2018-2023)

Year	Total counts (rates) per 100 000 of opioid-related overdose deaths			
	All adults	Hispanic adults	Non-Hispanic Black Adults	Non-Hispanic White Adults
2018	531 (11.99)	130 (15.56)	20 (9.28)	375 (11.77)
2019	605 (13.44)	140 (16.33)	41 (18.55)	411 (12.76)
2020	919 (20.76)	250 (28.48)	64 (28.30)	590 (18.76)
2021	1218 (26.65)	336 (37.82)	75 (37.15)	773 (24.14)
2022	1122 (24.24)	316 (34.84)	89 (43.33)	691 (21.53)
2023	1157 (25.00)	335 (36.94)	117 (56.96)	682 (21.25)

**Figure 1.**
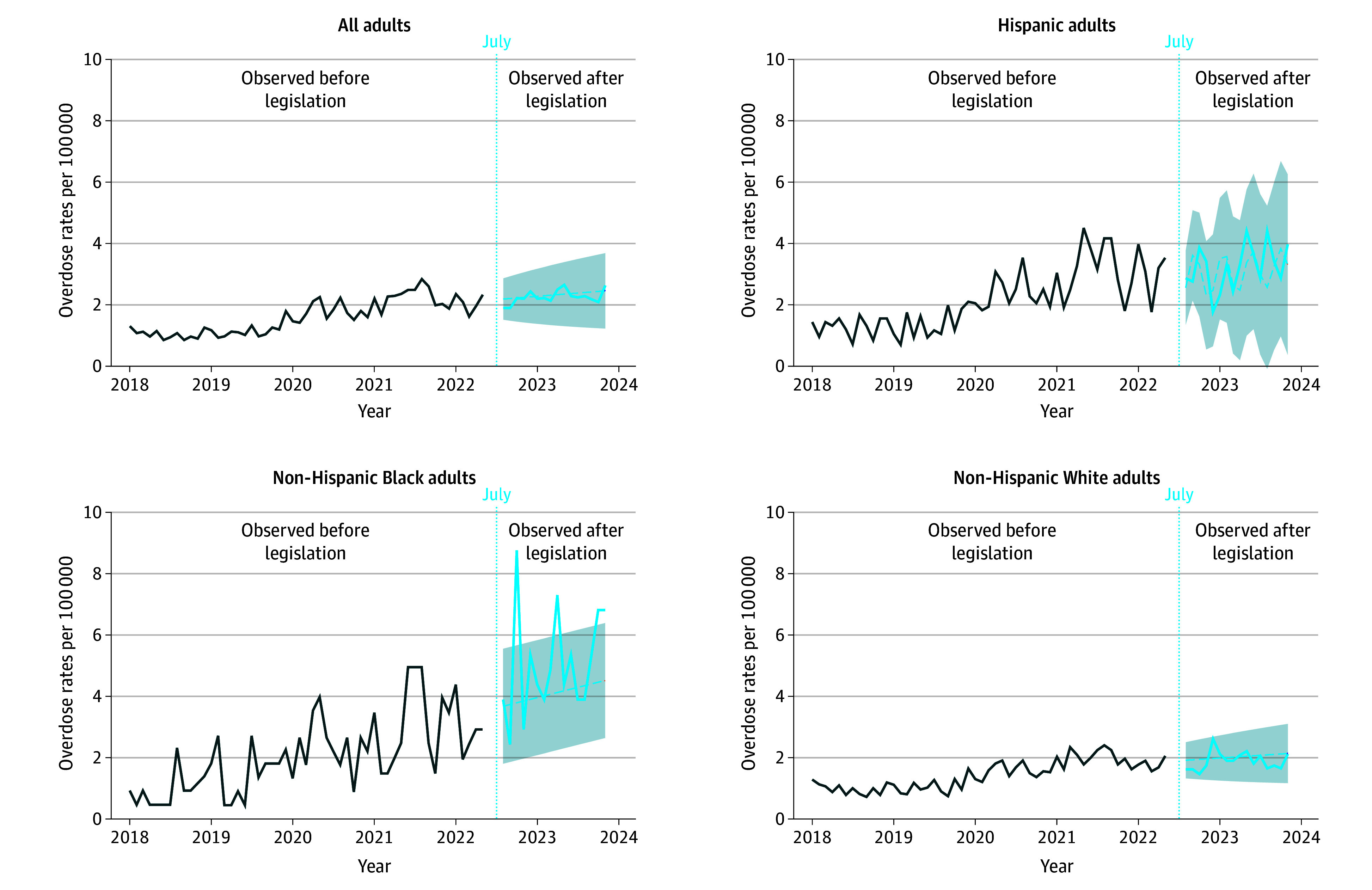
Forecasted and Observed Opioid-Related Overdose Rates in Colorado This analysis used autoregressive integrated moving average (ARIMA) models, displaying the monthly rate of opioid-related overdose deaths from January 2018 to November 2023 for the overall adult population (ARIMA, 0, 1, 1), Hispanic population (ARIMA, 3, 1, 2), non-Hispanic Black population (ARIMA, 0, 1, 1), and non-Hispanic White population (ARIMA, 0, 1, 1). The shading indicates 95% CIs; dashed lines, the mean forecast of opioid-related overdose deaths; the solid lines, the observed opioid-related overdose deaths; and the blue dotted line, the date the legislation increasing criminal penalties for fentanyl possession became effective.

**Figure 2.**
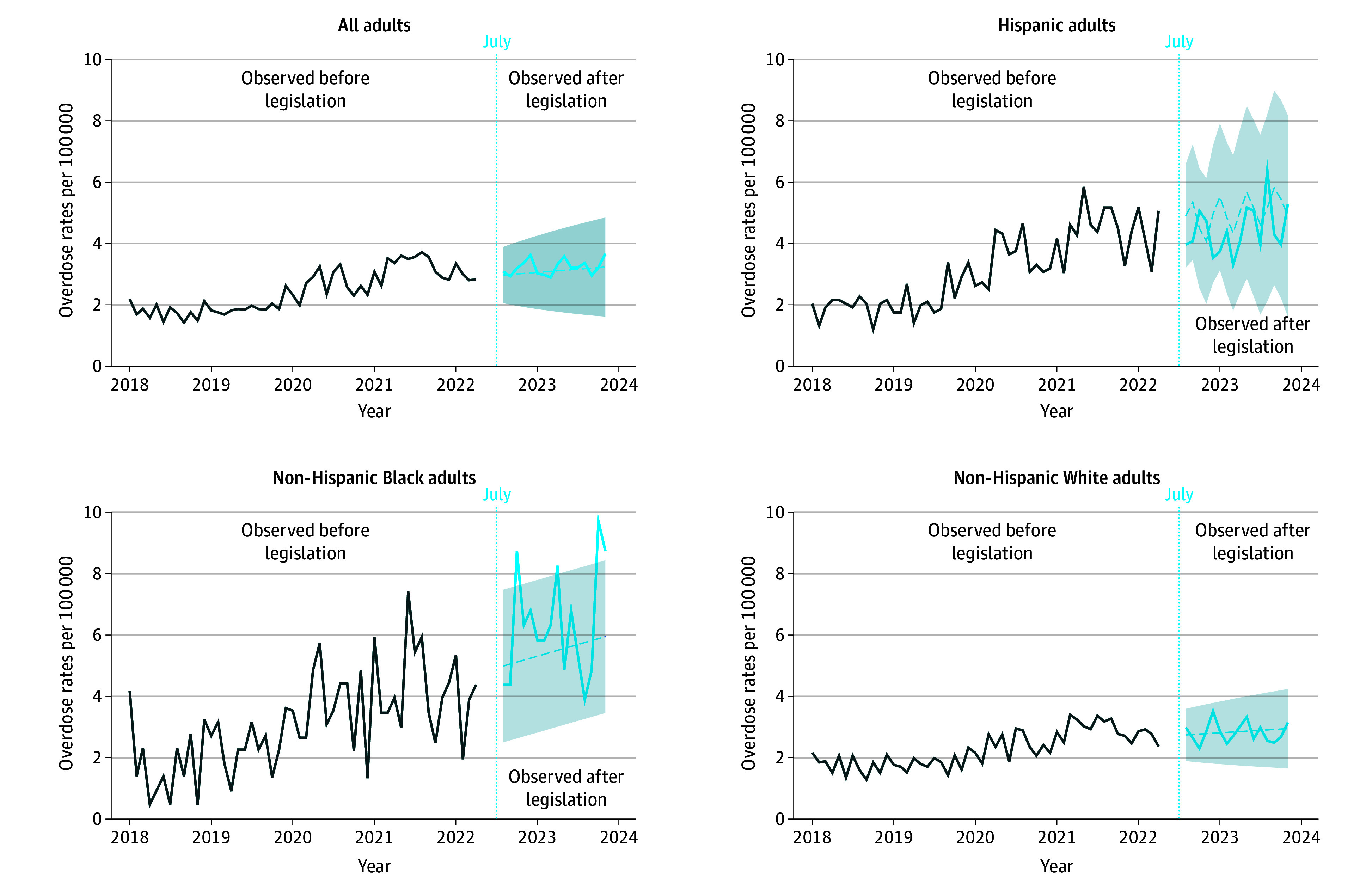
Comparison of Forecasted and Observed All-Drug Overdose Rates in Colorado This analysis used autoregressive integrated moving average (ARIMA) models, displaying the monthly rate of all-drug overdoses (sensitivity analysis) from January 2018 to November 2023 for the overall adult population (ARIMA, 0, 1, 1), Hispanic population (ARIMA, 3, 1, 2), non-Hispanic Black population (ARIMA, 0, 1, 1), and non-Hispanic White population (ARIMA, 0, 1, 1). The shading indicates 95% CIs; dashed lines, the mean forecast of opioid-related overdose deaths; the solid lines, the observed opioid-related overdose deaths; and the blue dotted line, the date the legislation increasing criminal penalties for fentanyl possession became effective.

In analyses including all OODs, the number of observed deaths did not significantly differ from the predicted number of OODs (Figure 1). In models stratified by race and ethnicity, observed OOD rates were similar to predicted rates for Hispanic and non-Hispanic White populations. For the non-Hispanic Black population, 4 of the 13 months after the legislation had observed OOD rates that were greater than predicted (October 2022, April 2023, October 2023, and November 2023; Figure 1). The largest deviation from predicted trends occurred among the non-Hispanic Black population in October 2022 (3.79 predicted to 8.76 observed deaths). Sensitivity analyses including all drug overdoses were similar to the results of the primary analysis (Figure 2).

## Discussion

Comparing observed with expected trends in OOD rates following the implementation of the 2022 bill to increase criminal penalties for fentanyl possession, we found no overall population-level changes. Subgroup analyses identified observed increases in OODs compared with what would be expected among the non-Hispanic Black population in Colorado. These findings are consistent with national trends of increasing OODs among Black populations.^7^

There has been a growing push to increase criminal penalties for drug possession and distribution. The goal for many of these policies is to address the overdose crisis. These results suggest that increasing criminal penalties for fentanyl possession did not decrease OODs, consistent with broader literature on criminal penalties for drug use from before the fentanyl era.^8^ For additional context, arrests for drug narcotic violations rose 5% from 2022 to 2024 in Colorado; however, this increase includes arrests for all controlled substances.^9^

In stratified analyses, the OOD rate was higher than expected among the non-Hispanic Black population in some months after the passage of HB 22-1326. Based on these findings, it is difficult to directly attribute months with higher-than-expected rates to the law’s passage. Future studies should further examine these trends. There have been increases in OODs among Black individuals in many states without similar legislation during the same period as this study. Black people are less likely to have access to harm-reduction services, medications for opioid use disorder, and support services for drug use than their White counterparts.^10^ Black communities have been disproportionately affected by punitive drug policies, experiencing higher rates of drug-related arrest and incarceration compared with White people, despite lower overall prevalence of drug use.^11^ Whether these historical experiences translate into a disproportionate impact of laws increasing criminal penalties for fentanyl possession on Black individuals remains an open question.

### Limitations

There are several limitations to this study. First, we cannot eliminate the possibility that other factors beyond HB 22-1326 were driving observed trends. The criminal provisions of the bill were implemented immediately in July 2022; however, most of the other provisions including increasing harm reduction and mandating that jails offer medications for opioid use disorders were implemented in February 2023. Second, we were unable to include individual-level or aggregate data on arrests, charges, or convictions for fentanyl possession, specifically, during the study period. As a result, we are unable to assess changes in enforcement patterns following the policy change or examine the extent to which criminal legal system involvement may have mediated observed trends in overdose deaths. Finally, we were unable to include a comparison group, making it difficult to assess any causal relationship between the law and OOD rates.

## Conclusions

Increasing criminal penalties for fentanyl possession was not associated with a positive or negative change in OODs in Colorado, an important finding for state government officials considering similar legislation. Further work is needed to understand the impact of these laws on other health outcomes such as initiation and retention on medications for opioid use disorder. Legislators should continue to focus on evidence-based strategies that increase access to naloxone and substance use treatment—especially methadone and buprenorphine, which have lagged for non-Hispanic Black communities.^12^

## Notes

Supplement.
Data Sharing Statement

## References

[1] Spencer M, Garnett M, Miniño A. Drug overdose deaths in the United States, 2002–2022. NCHS Data Brief, No. 491. National Center for Health Statistics; 2024. Accessed June 3, 2025. https://www.cdc.gov/nchs/products/databriefs/db491.htm.

[2] Tanz LJ, Gladden RM, Dinwiddie AT, . Routes of drug use among drug overdose deaths—United States, 2020-2022. MMWR Morb Mortal Wkly Rep. 2024;73(6):124-130. doi:10.15585/mmwr.mm7306a2 38358969 PMC10899081

[3] Fentanyl Accountability and Prevention Act, HB 22-1326, 2022 Regular Sess (Colo 2022). May 25, 2022. Accessed June 3, 2025. https://leg.colorado.gov/sites/default/files/2022a_1326_signed.pdf

[4] Schaffer AL, Dobbins TA, Pearson SA. Interrupted time series analysis using autoregressive integrated moving average (ARIMA) models: a guide for evaluating large-scale health interventions. BMC Med Res Methodol. 2021;21(1):58. doi:10.1186/s12874-021-01235-8 33752604 PMC7986567

[5] Helfenstein U. Box-Jenkins modelling in medical research. Stat Methods Med Res. 1996;5(1):3-22. doi:10.1177/096228029600500102 8743076

[6] Zoorob MJ, Park JN, Kral AH, Lambdin BH, Del Pozo B. Drug decriminalization, fentanyl, and fatal overdoses in Oregon. JAMA Netw Open. 2024;7(9):e2431612-e2431612. doi:10.1001/jamanetworkopen.2024.31612 39235814 PMC11378001

[7] Khan MR, Hoff L, Elliott L, . Racial/ethnic disparities in opioid overdose prevention: comparison of the naloxone care cascade in White, Latinx, and Black people who use opioids in New York City. Harm Reduct J. 2023;20(1):24. doi:10.1186/s12954-023-00736-7 36841763 PMC9959933

[8] More imprisonment does not reduce state drug problems. Pew Charitable Trusts. March 8, 2018. Accessed June 3, 2025. https://www.pew.org/en/research-and-analysis/issue-briefs/2018/03/more-imprisonment-does-not-reduce-state-drug-problems

[9] DUI/Drugs 2024 Colorado. Colorado Crime Statistics. Accessed May 8, 2025. https://coloradocrimestats.state.co.us/tops/report/drugs-dui/colorado/2024

[10] Lopez AM, Thomann M, Dhatt Z, . Understanding racial inequities in the implementation of harm reduction initiatives. Am J Public Health. 2022;112(S2):S173-S181. doi:10.2105/AJPH.2022.306767 35349311 PMC8965181

[11] Bailey ZD, Krieger N, Agénor M, Graves J, Linos N, Bassett MT. Structural racism and health inequities in the USA: evidence and interventions. Lancet. 2017;389(10077):1453-1463. doi:10.1016/S0140-6736(17)30569-X 28402827

[12] Lagisetty PA, Ross R, Bohnert A, Clay M, Maust DT. Buprenorphine treatment divide by race/ethnicity and payment. JAMA Psychiatry. 2019;76(9):979-981. doi:10.1001/jamapsychiatry.2019.0876 31066881 PMC6506898

