# Supplementary material for: Implementation of a Statewide Fentanyl Possession Law and Opioid-Related Overdose Deaths
Source: JAMA Health Forum. 2025 Aug 1;6(8):e252654. doi: 10.1001/jamahealthforum.2025.2654 (PMC12317351; doi:10.1001/jamahealthforum.2025.2654)
Supplement: Supplement. — Data Sharing Statement [file jamahealthforum-e252654-s001.pdf]

Data Sharing Statement

Jurecka. Implementation of a Statewide Fentanyl Possession Law and Opioid-Related Overdose Deaths. *JAMA Health Forum*. Published August 01, 2025. doi:10.1001/jamahealthforum.2025.2654

Data
Data available: No
